# In Vitro Study of the Bioavailability and Bioaccessibility of the Main Compounds Present in Ayahuasca Beverages

**DOI:** 10.3390/molecules26185555

**Published:** 2021-09-13

**Authors:** Joana Gonçalves, Miguel Castilho, Tiago Rosado, Ângelo Luís, José Restolho, Nicolás Fernández, Eugenia Gallardo, Ana Paula Duarte

**Affiliations:** 1 Centro de Investigação em Ciências da Saúde (CICS-UBI), Universidade da Beira Interior, Av. Infante D. Henrique, 6200-506 Covilhã, Portugal; joanadgoncalves13@gmail.com (J.G.); miguel.castilho@ubi.pt (M.C.); tiagorosadofful@hotmail.com (T.R.); jrestolho@gmail.com (J.R.); 2 Laboratório de Fármaco-Toxicologia, UBIMedical, Universidade da Beira Interior, Estrada Municipal 506, 6200-284 Covilhã, Portugal; 3 Cátedra de Toxicología y Química Legal, Laboratorio de Asesoramiento Toxicológico Analítico (CENATOXA), Facultad de Farmacia y Bioquímica, Universidad de Buenos Aires, Junín 956, Ciudad Autónoma de Buenos Aires (CABA), Buenos Aires C1113AAD, Argentina; nfernandez@ffyb.uba.ar

**Keywords:** ayahuasca, bioavailability, bioaccessibility, PAMPA, HPLC-DAD

## Abstract

Ayahuasca is a psychoactive beverage that contains the psychoactive compound *N*,*N*-dimethyltryptamine and *β*-carboline alkaloids. This study aims at determining in vitro the bioavailability and bioaccessibility of the main compounds present in decoctions of four individual plants, in a commercial mixture and in four mixtures of two individual plants used in the preparation of Ayahuasca. The samples were subjected to an in vitro digestion process, and the Caco-2 cell line was used as an absorption model. The integrity and permeability of the cell monolayer were evaluated, as well as the cytotoxicity of the extracts. After digestion and cell incubation, the compounds absorbed by the cell monolayer were quantified by high-performance liquid chromatography coupled to a diode array detector. The results showed that compounds such as *N*,*N*-dimethyltryptamine, Harmine, Harmaline, Harmol, Harmalol and Tetrahydroharmine were released from the matrix during the in vitro digestion process, becoming bioaccessible. Similarly, some of these compounds, after being incubated with the cell monolayer, were absorbed, becoming bioavailable. The extracts did not show cytotoxicity after cell incubation, and the integrity and permeability of the cell monolayer were not compromised.

## 1. Introduction

Ayahuasca is a psychoactive beverage traditionally consumed in the Amazon Basin of South America [1]. This word of Quechua origin, means “vine of the soul” or “vine of the dead” and is composed of the terms “*aya*” and “*wasca*”, which means “spirit” and “vine”, respectively [2,3]. This psychoactive beverage consists of a thick, oily and brownish decoction, which is prepared from the leaves of *Psychotria viridis* (*P. viridis*) and scraps from the stem of *Banisteriopsis caapi* (*B. caapi*) [4,5]. However, over the years, the preparation of Ayahuasca has undergone variations [1,2,6]. Thus, some species of natural origin have been used in the preparation of the beverage, namely *Brunfelsia* spp., *Daturaolens*, *Malouetia tamarquina*, *Psychotria carthagenesis*, *Brugmansia suaveolens*, *Tabernaemontana* spp., or *Nicotiana tabacum*, replacing *P. viridis*, or *Peganum harmala*, Harmine freebase/HCl, Moclobemide and Tetrahydroharmine freebase/HCl, replacing *B. caapi* [6,7].

**Figure 1:**
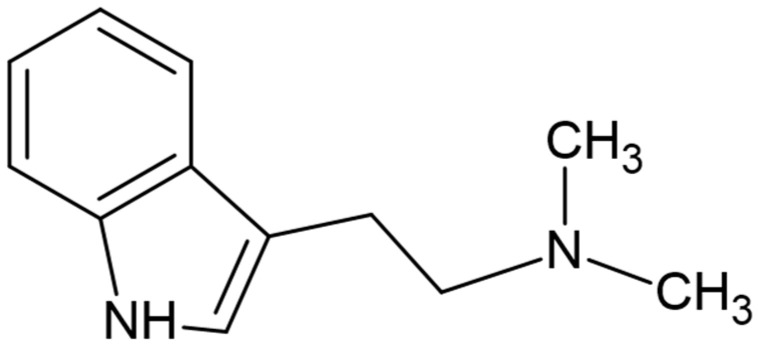
Molecular structure of DMT.

This psychoactive beverage was traditionally used by native healers for divine cults and in the cure of psychological disorders, stimulation of visual creativity and creative thinking [1,6]. Its hallucinogenic effects are due to the presence of N,N-dimethyltryptamine (DMT) (Figure 1) from *P. viridis*, which behaves as an agonist of serotonin receptors (5-HT1A/2A/2C) [3]. When ingested alone, this compound is inactive, as it is rapidly metabolized by peripheral monoamine oxidase A (MAO-A) [8]. However, in the presence of β-carbolinic alkaloids, such as Harmaline, Harmine and Tetrahydroharmine (THH), from *B. caapi*, DMT can access the central nervous system, since these are temporary inhibitors of hepatic and intestinal MAO-A [2]. THH also acts as a serotonin reuptake inhibitor, enhancing the effects of DMT [2,3]. The knowledge about this synergy between compounds present in the two plants has been known by indigenous peoples for about 3000 years [9].

Although Ayahuasca has been consumed for centuries, in the last 25 years, its use has been increasing in different parts of the world [10,11]. The consumption of Ayahuasca in controlled environments such as religious rituals and experimental procedures is not associated with psychotic episodes [10]. However, the expansion of Ayahuasca has raised some concerns about the possible adverse effects associated with consumption, but also an interest in its potential therapeutic effects [6,10].

Bioavailability and bioaccessibility are important concepts that make it possible to understand the behaviour of some compounds in the body. Bioaccessibility consists of the amount of a compound that is released from a matrix, being available to be absorbed, after ingestion and consequent digestion [12]. On the other hand, bioavailability is defined as the fraction that reaches the bloodstream and that, after metabolization and distribution, produces an effect [13]. In vitro digestion is a procedure that has been used to determine the fraction of compounds that are released from the matrix and become bioaccessible [14]. In vitro digestion models aim to mimic the digestive process along the digestive tract (mouth, stomach and intestine), simulating physiological conditions such as pH, salt concentration, digestion time, among others [15,16]. Regarding the assessment of bioaccessibility, cell lines are frequently used, namely the line derived from a human colon carcinoma Caco-2, due to its similar morphology with the cells of the small intestine [17]. In addition, these cells have narrow intracellular junctions and express enzymes similar to those that are present in the small intestine, allowing to mimic the transport mechanisms that occur therein [18,19,20].

There are no studies concerning the fate of the active ingredients of Ayahuasca formulations after ingestion, namely concerning their absorption to the general circulation for distribution. Therefore, with this study we aimed at evaluating the bioavailability and bioaccessibility of the active compounds present in four individual plants, a commercial mixture and four plant mixtures, used to prepare the Ayhauasca decoction. For that, an in vitro digestion process was used, as well as the parallel artificial membrane permeability assay (PAMPA) using Caco-2 cells, in order to know better their path in the human body and part of the mechanisms regulating their passage to the blood stream.

## 2. Results and Discussion

Considering the several potential bioactive properties of Ayahuasca, four individual decoctions of each plant used in the preparation of Ayahuasca were prepared in this work, as well as four decoctions of a mixture of plants (with two different plant materials, one source of DMT and the other of β-carboline alkaloids). A decoction of a commercial mixture was also prepared. The bioavailability and bioaccessibility of the main compounds present in Ayahuasca was evaluated in the nine samples.

### 2.1. Characterization of Main Compounds in Initial Samples and after Digestive Process

DMT is present in some plants used in the preparation of Ayahuasca beverages. Given the use of plant samples containing this compound in religious rituals, it has received some attention over the years due to its psychoactive effects [21]. Besides that, the access of this compound to the bloodstream is dependent on the β-carboline alkaloids [1,8,22,23]. Thus, an analytical method by high performance liquid chromatography coupled diode array detector (HPLC-DAD) was developed, which allowed the quantification of the main active compounds present in the samples of Ayahuasca beverages (Table 1). This analytical method was developed and validated in accordance with the standards of the Food and Drug Administration [24]. Thus, it was linear between 0.16 and 10.00 μg/mL for Harmol, THH, Harmaline and Harmine, between 0.31 and 10.00 μg/mL for Harmalol and between 0.031 and 1.00 μg/mL for DMT, with coefficients of determination (R^2^) higher than 0.997. The intra- and inter-day precision revealed coefficients of variation below 15% and the accuracy was within the range of ±15%. The LOD and LLOQ obtained were 0.31 μg/mL for all compounds, except for DMT (0.031 μg/mL).

All samples from a mixture of two plants showed substantial concentrations of DMT, with the mixture of *M. hostilis* and *B. caapi* having the highest concentration, and the mixture of *P. viridis* and *B. caapi* having the lowest concentration. Regarding the individual samples, both the *P. viridis* and *M. hostilis* decoctions and the commercial mixture showed substantial amounts of DMT. In contrast, in the decoctions of *B. caapi* and *P. harmala*, this compound was not detected. Moreover, all mixtures presented considerable concentrations of β-carboline alkaloids, with the mixture of *M. hostilis* and *P. harmala* presenting the highest amount. Regarding the individual samples, these compounds were not detected in the decoctions of *P. viridis* and *M. hostilis*. On the other hand, in the decoctions of *B. caapi* and *P. harmala*, all β-carboline alkaloids were detected, with THH and Harmol being in greater quantity in *B. caapi* and Harmine, Harmalol and Harmaline in greater quantity in *P. harmala*. Regarding the commercial mixture, it was possible to detect all β-carbolines, except for Harmalol. Bensalem et al. [25] carried out the quantification of Harmine, Harmaline, Harmol and Harmalol in samples of *P. harmala*, having verified that, similarly to what was observed in the present study, the compound with the highest concentration was Harmaline, followed by Harmine, Harmalol and, the least concentrated, Harmol. In addition, Avula et al. [26] carried out the quantification of Harmol, Harmine, Harmaline, among other compounds, using ultra-performance liquid chromatography-triple quadrupole mass spectroscopy with ultraviolet detection (UPLC-UV-MS) and high-performance thin-layer chromatography (HPTLC). It was found that, similarly to the results now obtained, Harmine was found in a higher quantity than Harmol, being Harmaline not detected [26]. Several studies were also carried out, with the aim of determining the concentration of DMT and β-carbolines in Ayahuasca samples. Pires et al. [27] used gas chromatography equipment with nitrogen/phosphorous detector to quantify DMT, Harmine, Harmaline and THH in eight samples of Ayahuasca. Similar to what was observed in the present work, the four compounds were detected in all samples [27]. Moreover, Souza et al. [28] analysed 38 Ayahuasca samples using liquid chromatography coupled to tandem-mass spectrometry (LC-MS/MS) verified the presence of THH, DMT, Harmine and Harmaline. Recently, Chambers et al. [29] quantified the DMT present in 6 samples of Ayahuasca, obtaining values between 45.7 and 230.5 mg/L. It is important to point out that the concentrations of each compound in the Ayahuasca samples can be very variable. This fact can be due to a number of factors, namely the variability of the proportion used by each user, as well as the different preparation methods [27,28]. Additionally, the concentration of the compounds in each plant can also be very variable [27]. According to Kaasik et al. [30], the average variations of concentrations of DMT, THH, Harmine and Harmaline, can be, respectively, 26.2%, 29.8%, 41.5% and 2.5%. The samples used in this study were acquired online, making it difficult to know their degree of purity.

After quantifying the main compounds present in samples of Ayahuasca beverages, the same compounds were quantified over the three stages of the in vitro digestion process (salivary, gastric and duodenal). By observing the aliquots collected in each step, it is possible to verify that there were colour variations throughout the process. Likewise, the concentrations of DMT and β-carboline alkaloids also varied between samples and, within the same sample, between digestion steps (Table 2).

**Figure 2:**
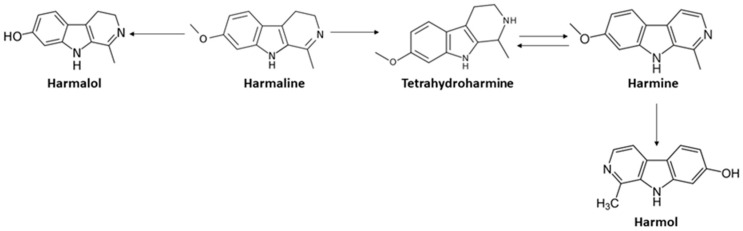
Reactions between β-carboline alkaloids.

Analysing the results, it was possible to verify that the amount of DMT varies throughout the in vitro digestion process. In general, the concentration of DMT at the end of the entire process decreased in samples of *M. hostilis*, in the commercial mixture and in the mixtures of *M. hostilis* and *B. caapi* and *M. hostilis* and *P. harmala*. Conversely, there was an increase in DMT in the sample of *P. viridis*, while in the mixtures of *P. viridis* and *B. caapi* and *P. viridis* and *P. harmala* there were no noticeable changes. With respect to β-carbolines, there was a variation from compound to compound. The concentration of Harmol remained constant throughout the digestion process of the sample of *M. hostilis* and *B. caapi*, increased in the mixture of *P. viridis* and *B. caapi* and was not detected in the other samples. It was also not possible to detect Harmalol during the digestion of the samples of *B. caapi* and in the mixture of *M. hostilis* and *B. caapi*. In the other samples where this compound was initially detected, its concentration decreased slightly. Regarding THH, it was verified that its concentration increased in the samples of *B. caapi* and decreased in the commercial mixture and in mixtures of *P. viridis* and *B. caapi* and *M. hostilis* and *P. harmala*. A slight decrease of this compound was also observed in the sample of *P. harmala* and in the mixture of *M. hostilis* and *B. caapi*. In the mixture of *P. viridis* and *P. harmala* this compound was not detected. It was verified that the concentration of Harmine increased, except in the commercial mixture (not detected) and in *P. harmala* (decreased). The concentration of Harmaline remained constant in the mixture of *M. hostilis* and *B. caapi* and decreased in the sample of *P. harmala*, in the commercial mixture and in the mixture of *P. viridis* and *P. harmala*. In the samples of *B. caapi*, and in the mixtures of *P.viridis* and *B. caapi* and *M. hostilis* and *P. harmala*, there was a slight increase in the concentration of Harmaline. These variations in the concentrations of β-carboline alkaloids may be due to the fact that these compounds degrade and easily give rise to another β-carboline (Figure 2) [31].

So far, no bioaccessibility studies have been carried out on Ayahuasca or plants used in its preparation. Digestion studies including this type of samples have not been carried out so far, so it is not possible to make a comparison.

### 2.2. Cell Culture

#### 2.2.1. Evaluation of Cell Viability

The cytotoxicity of each sample was assessed using the MTT assay. In analysing the results, it was verified that there was a slight decrease in cell viability in the samples of the digested commercial mixture and in the crude extract of *B. caapi*. The other samples showed no decrease in cell viability (Table 3). These results are in agreement with those obtained by Katchborian-Neto et al. [32], which evaluated the cytotoxicity of Ayahuasca samples in SH-SY5Y cells. Additionally, three of the samples intensely increased cell viability within the first 48 h [32]. In addition, Samoylenko et al. [33] evaluated the cytotoxicity of *B. caapi* extracts in six cell lines, verifying that the extracts did not show cytotoxicity.

#### 2.2.2. Evaluation of the Electrical Resistance of the Cell Transendothelial Membrane

The integrity of the cell monolayer was evaluated by the TEER assay, before and after cell incubation with the extracts (Table 4). The TEER assay allows monitoring the integrity of cell layers in in vitro assays, as well as possible changes in intercellular junctions, by evaluating transendothelial electrical resistance [34]. Analysing the results of the TEER measurements before incubation with the extracts, it was observed that the monolayer was intact, since the values were above the 150−200 Ω cm^2^ range, minimum acceptable limit [35]. After incubation with the extracts, a new TEER measurement was performed, with no significant differences between the values of the first and second measurements. Additionally, the values of the second measurement were also above the minimum acceptable limit. Therefore, the integrity of the cell monolayer was confirmed [35]. So far, there are no studies with Ayahuasca samples where the TEER assay has been performed.

#### 2.2.3. Evaluation of Cell Monolayer Permeability

Cell monolayer permeability was assessed by Lucifer Yellow permeability assay (Table 5). The Lucifer Yellow permeability assay allows evaluating the permeability characteristics of a cell monolayer, by measuring the passive diffusion of different molecules through it [36]. This assay was performed after exposing the cells to extracts. Analysing the results, it was shown that there were no significant changes in cell permeability, when compared to the control. These results are in agreement with those obtained in the TEER assay, suggesting that there were no changes in intracellular spaces, nor in cell barrier function and in membrane permeability [37,38]. Previous studies also suggest that both TEER measurement and permeability are related, and that a significant increase in the permeability is accompanied by a decrease in TEER values [37,38]. Similarly to what was observed in the TEER assay, no studies were found where the Lucifer Yellow permeability assay was performed with Ayahuasca samples.

#### 2.2.4. Characterization of the Main Compounds after Cell Incubation

The amount of compounds present in the collected aliquots after cell incubation of crude and digested extracts were also quantified by HPLC-DAD (Table 6 and Table 7). It was verified that DMT, Harmine and Harmaline are the compounds, from those present in the digested extract, which cross the cell monolayer the most. Similarly, in the crude extract the same results were observed. The concentration of these three compounds in all samples and for both extracts, increased gradually in the basolateral compartment throughout the incubation period, except in the digested extract in the mixture of *P. viridis* and *B. caapi*, where DMT increases after 2 h of incubation, remaining approximately constant until 4 h of cell incubation. In general, in the digested extract, all the compounds gradually increased during cell incubation, except for Harmol and Harmalol, which were not detected during the entire process. Similarly, in the crude extract, Harmalol was not detected, but Harmol was detected after 2 h of incubation in the *P. harmala* sample. Moreover, as in what was observed in the digested extract, in the crude extract all compounds gradually increased during cell incubation, except for the THH present in the mixture of *M. hostilis* and *B. caapi*, which decreases slightly after 2 h of incubation, increasing again after 4 h.

In general, it was possible to observe that all the analysed compounds managed to cross the cell monolayer, except Harmalol and Harmol. In the digested samples the bioavailability percentages ranged between 8.30–28.9% for DMT, 0–29.63% for Harmaline and 33.03–57.58% for Harmine. So far, no studies have been carried out on the bioavailability of Ayahuasca, so it is not possible to make comparisons with the present study. However, differences in β-carboline concentrations can be explained by the rapid mutual conversion of these compounds (Figure 2) [31]. Additionally, DMT easily crosses the barriers of the body, since it is a small and hydrophobic molecule with a low molecular weight [21]. It was also observed that the amount of the compounds decreased after crossing the cell monolayer, when compared to the values obtained after in vitro digestion. This fact has already been verified in bioavailability studies with other compounds of natural origin [39,40]. In a study that evaluated the bioavailability and bioaccessibility of *Prunus avium* L., carried out by our research group, this same decrease in the amount of compounds after crossing the Caco-2 cell monolayer was also verified [14].

## 3. Materials and Methods

### 3.1. Chemicals and Materials

The analytical standards DMT, Harmine, Harmaline, THH, Harmol and Harmalol were kindly provided by Nal von Minden, GmbH (Regensburg, Germany). Lucifer Yellow, 3-[4,5-dimethylthiazol-2-yl]-2,5-diphenyltetrazolium bromide (MTT) and Roswell Park Memorial Institute (RPMI) medium were obtained from Sigma-Aldrich (Sintra, Portugal). Methanol (HPLC grade) was obtained from Fischer Chemical (Loughborough, UK). Formic acid and dimethyl sulfoxide (99.9% of purity) were purchased from Sigma-Aldrich (Sintra, Portugal). Deionized water was obtained from a Milli-Q System (Millipore, Billerica, MA, USA).

### 3.2. Sample and Working Solutions Preparation

All vegetal samples were acquired online from Shayana Shop (https://www.shayanashop.com, Amsterdam, The Netherlands) (accessed on 25 May 2019). The decoctions of Ayahuasca were prepared according to a traditional recipe kindly provided by Dr. Nicolás Fernández. Thus, 0.210 g of each of the five vegetal samples were weighed (*P. viridis* (leaves), *P. harmala* (seeds), *B. caapi* (scraps from the stem), *M. hostilis* (root bark) and commercial mixture) and were then macerated in a mortar with a few drops of water. After that, 250 mL of ultra-pure water was added, and the mixture was transferred to a Schott flask. This preparation was boiled at 100 °C for 4 h. Similarly, four decoctions were prepared where two of the above vegetal samples were mixed (*P. viridis* and *P. harmala*; *P. viridis* and *B. caapi*; *M. hostilis* and *P. harmala*; *M. hostilis* and *B. caapi*). After boiling, the samples were cooled, filtered, frozen at −80 °C and freeze-dried.

Individual stock solutions of DMT, Hamine, Harmaline, Harmol and Harmalol were prepared at 1 mg/mL in methanol. Working solutions were prepared by serial dilutions in methanol.

### 3.3. In Vitro Simulation of Human Digestion Process

The in vitro digestion assay was carried out as described in a previous work [14]. Initially, salivary fluid (potassium chloride, monosodium phosphate, sodium sulphate, sodium chloride, sodium bicarbonate, urea, α-amylase, mucin and uric acid), gastric fluid (sodium chloride, monosodium phosphate, potassium chloride, calcium chloride, ammonium chloride, hydrochloric acid, glucose, urea, pepsin, mucin and bovine serum albumin), duodenal fluid (sodium chloride, sodium bicarbonate, potassium dihydrogen phosphate, potassium chloride, magnesium chloride, hydrochloric acid, urea, calcium chloride dihydrate, bovine serum albumin, pancreatin and lipase) and bile fluid (sodium chloride, sodium bicarbonate, potassium chloride, hydrochloric acid, urea, calcium chloride dihydrate, bile and bovine serum albumin) were prepared. For the assay, each freeze-dried decoction was dissolved in 100 mL of deionized water. To each of the nine samples, 6 mL of simulated salivary fluid (pH 6.8) was added, being this mixture was incubated at 37 °C for 5 min with orbital shaking at 90 rpm. Then, 12 mL of simulated gastric fluid (pH 1.3) was added, followed by incubation in the same conditions for 2 h. After this time, 6 mL of simulated bile fluid (pH 8.2), 12 mL of simulated duodenal fluid (pH 8.1) and 2 mL of sodium bicarbonate solution (1M) was added. The solution was incubated again at 37 °C with orbital shaking at 90 rpm for 2 h.

Aliquots were collected at the end of each stage of the in vitro digestion process, which were immediately cooled, then frozen at −80 °C for 30 min and later sonicated at 4 °C in an ice bath for 30 min. Subsequently, the samples were filtered through a 0.22 μm cellulose acetate pore filter and subsequently analysed by high-performance liquid chromatography (HPLC). Furthermore, an additional aliquot of the last stage of the in vitro digestion of each sample was collected, which was also placed on ice, frozen at −80 °C and sonicated. Then, their pH was measured and corrected, when necessary, to physiological pH. Subsequently, these aliquots were used in the PAMPA assay.

### 3.4. Cell Culture

The Caco-2 cell line (Database name: American Type Culture Collection (ATCC) Accession numbers: HTB-37)[41] was cultured in RPMI medium supplemented with 1% antibiotic mixture and 10% foetal bovine serum, at passages between 33 and 37. Subsequently, the cells were incubated at 37 °C in a humidified atmosphere containing 5% CO_2_.

For the MTT assay, the cells were seeded in 96 multi-well plates (cat. number 734-2802 avantor, VWR, Amadora Portugal) at a cell density of 0.5 × 10^4^. For the PAMPA assay, the cells were seeded in culture inserts, placed in 12 multi-well plates (cat. number 734-2731 avantor, Laborspirit, Santo Antão de Tojal, Portugal) at a cell density of 6 × 10^4^, remaining for a period of 21 days in order to form a confluent monolayer. After that time, 500 μL of each of the nine samples (digested and undigested) was added to the apical chamber, to be in contact with the cell monolayer (*P. viridis*—0.278 mg/mL; *B. caapi*—0.062 mg/mL; *P. harmala*—0.226 mg/mL; *M. hostilis*—0.382 mg/mL; commercial mixture—0.156 mg/mL; *P. viridis* + *B. caapi*—0.203 mg/mL; *P. viridis* + *P. harmala*—0.344 mg/mL; *M. hostilis + B. caapi*—0.555 mg/mL e *M. hostilis* + *P. harmala*—0.4 mg/mL). After 1, 2 and 4 h, 250 μL was collected in the basolateral chamber. The collected aliquots were analysed by HPLC. All tests were performed in triplicate.

#### 3.4.1. MTT Cell Viability Assay

The cytotoxicity of the samples was assessed by the MTT assay. For that, after the cells became confluent, they were exposed to the samples (digested and undigested) 1, 2 and 4 h. RPMI medium was used as a negative control. After incubation, the medium was removed and an MTT solution was added. Then, the cells were incubated for 3 h. After that time, the MTT solution was removed, and the formazan crystals formed were dissolved in dimethyl sulfoxide (DMSO), being the absorbance measured using a microplate reader at 570 nm.

#### 3.4.2. Transepithelial Electrical Resistance Assay

The integrity of the cell monolayer was evaluated by measuring the transepithelial electrical resistance (TEER). Before the incubation of the cells’ monolayer with the extracts (digested and undigested), the TEER was measured. Initially, the electrode of the transepithelial resistance meter (EVOM2, World Precision Instrument, Sarasora, FL, USA) was equilibrated with RPMI medium and then was placed in each well to form an angle of 90°. The procedure was performed in triplicate and the TEER was determined according to the following equation:

TEER value = (mean of the resistances of each well − mean of the resistance of blank) × insert area(1)

#### 3.4.3. Lucifer Yellow Permeability Assay

The Lucifer Yellow Permeability Assay allows evaluating changes in the permeability characteristics of the cell monolayer after passive passage of compounds. This test was performed as described in a previous work [14]. Briefly, the RPMI medium of the chambers delimited by the insert (apical and basolateral) was removed and replaced by 500 μL of the Lucifer Yellow solution in the apical chamber and 1.5 mL of Hank’s balanced salt solution (HBSS) in the basolateral chamber. After that, the multi-well was incubated for 1 h, and then 200 μL of each basolateral chamber was pipetted to another culture plate, being the fluorescence measured at 485 nm (excitation) and 535 nm (emission) using a spectrofluorimeter. HBSS was used as a blank and a Lucifer Yellow solution (0.1 mg/mL) was used as a positive control. The permeability percentage was calculated as follows:

% permeability = (mean of fluorescence of each well − fluorescence of blank)/(fluorescence of positive control − fluorescence of blank) × 100(2)

### 3.5. Instrumental and Chromatographic Conditions

The quantification of main compounds present in Ayahuasca beverages was performed on an HPLC system coupled to a diode array detector (DAD) (Agilent technologies Soquímica, Lisbon, Portugal). The mobile phase was composed of 0.1% formic acid in methanol (A) and 0.1% formic acid in water (B). The elution was carried out in gradient mode and included 5% A (0–2 min), 50% A (2–32 min) and again, 5% A (32–40 min). The flow rate was 1.5 mL/min, and the injection volume was 50 μL. The stationary phase consisted of an YMC-Triart PFP (5 μm, 4.6 i.d. × 150 mm) analytical column coupled to a Guard-c holder (4 × 10 mm) and a Triart PFP (5 μm, 3 ×10 mm) pre-column, all from YMC Europe GMBH (Solítica, Lisbon, Portugal), being maintained at 25 °C. Harmine and Harmol were detected at 246 nm, DMT and THH at 278 nm and Harmaline and Harmalol at 360 nm. The temperature of the sampler was set at 4 °C.

### 3.6. Statistical Analysis

The results are expressed as mean values with standard deviations (SD). The Student’s *t*-test was employed and statistically significant values were considered when *p* < 0.05 (*).

## 4. Conclusions

During the in vitro digestion, the compounds were released from the matrix, becoming bioaccessible. The concentration of β-carboline alkaloids shows an appreciable transformation, while the variation of DMT is smaller. After the in vitro digestion, the detected compounds could be absorbed by the cell monolayer, becoming bioavailable but in lower concentrations. Likewise, the compounds present in the extracts that did not undergo in vitro digestion also became bioavailable. So, it can be inferred that digestion is not essential to occur absorption at the intestine level.

After cell incubation with the extracts, it was verified that they were not cytotoxic, and the integrity and the permeability of the cell monolayer remained unchanged, suggesting that the compounds did not interfere with intercellular junctions.

Further studies in which a more realistic approximation of the intestinal matrix is used should be carried out in order to overcome possible flaws of the used model.

## Figures and Tables

**Table 1:** Concentration of the main compounds of ayahuasca in different vegetal samples. The values are expressed as mean (µg/mg extract) ± SD.

Samples	Compounds	Initial Concentrations
*P. viridis*	DMT	6.50 ± 0.01
*B. caapi*	THH	5.00 ± 0.10
	Harmol	0.14 ± 0.00
	Harmine	10.00 ± 0.28
	Harmalol	0.05 ± 0.00
	Harmaline	4.68 ± 0.14
*P. harmala*	THH	3.05 ± 0.04
	Harmol	0.02 ± 0.00
	Harmine	12.00 ± 0.00
	Harmalol	0.66 ± 0.01
	Harmaline	17.00 ± 0.00
*M. hostilis*	DMT	10.50 ± 0.02
Commercial mixture	DMT	10.40 ± 0.01
	THH	2.09 ± 0.07
	Harmol	0.01 ± 0.00
	Harmine	0.02 ± 0.00
	Harmalol	ND
	Harmaline	0.37 ± 0.02
*P. viridis* + *B. caapi*	DMT	4.50 ± 0.01
	THH	2.50 ± 0.07
	Harmol	0.01 ± 0.00
	Harmine	0.48 ± 0.00
	Harmalol	ND
	Harmaline	0.07 ± 0.00
*P. viridis* + *P. harmala*	DMT	6.50 ± 0.09
	THH	0.63 ± 0.05
	Harmol	0.02 ± 0.00
	Harmine	0.30 ± 0.01
	Harmalol	0.08 ± 0.00
	Harmaline	0.48 ± 0.01
*M. hostilis* + *B. caapi*	DMT	8.00 ± 0.02
	THH	1.90 ± 0.06
	Harmol	0.03 ± 0.00
	Harmine	0.82 ± 0.02
	Harmalol	0.04 ± 0.00
	Harmaline	0.12 ± 0.00
*M. hostilis* + *P. harmala*	DMT	8.50 ± 0.01
	THH	3.44 ± 0.05
	Harmol	0.06 ± 0.00
	Harmine	9.00 ± 0.00
	Harmalol	0.36 ± 0.00
	Harmaline	13.5 ± 0.06

ND-not detected.

**Table 2:** Concentration of the main compounds of ayahuasca in different digestion steps. The values are expressed as mean (µg/mL) ± SD.

Samples	Compounds	Salivary	Gastric	Duodenal
*P. viridis*	DMT	0.84 ± 0.60	7.77 ± 0.08	7.49 ± 0.19
*B. caapi*	THH	0.83 ± 0.00	0.78 ± 0.13	1.05 ± 0.09
	Harmol	ND	ND	ND
	Harmine	1.56 ± 0.00	4.13 ± 0.03	1.98 ± 0.03
	Harmalol	ND	ND	ND
	Harmaline	0.19 ± 0.00	0.33 ± 0.00	0.21 ± 0.00
*P. harmala*	THH	1.52 ± 0.09	1.66 ± 0.12	1.32 ± 0.01
	Harmol	ND	ND	ND
	Harmine	18.38 ± 0.18	19.52 ± 0.05	10.02 ± 0.01
	Harmalol	1.47 ± 0.04	1.54 ± 0.02	1.03 ± 0.07
	Harmaline	29.66 ± 0.10	26.18 ± 0.14	22.88 ± 0.26
*M. hostilis*	DMT	9.55 ± 0.03	8.96 ± 0.17	8.33 ± 0.00
Commercial mixture	DMT	4.28 ± 0.05	4.09 ± 0.02	3.38 ± 0.08
	THH	0.95 ± 0.05	1.42 ± 0.00	0.50 ± 0.03
	Harmol	ND	ND	ND
	Harmine	ND	ND	ND
	Harmalol	1.21 ± 0.00	1.02 ± 0.01	0.81 ± 0.01
	Harmaline	1.29 ± 0.03	1.12 ± 0.01	0.85 ± 0.00
*P. viridis* + *B. caapi*	DMT	2.37 ± 0.01	1.61 ± 0.05	2.00 ± 0.02
	THH	3.33 ± 0.05	4.05 ± 0.11	2.82 ± 0.02
	Harmol	0.29 ± 0.00	0.34 ± 0.00	0.26 ± 0.01
	Harmine	1.23 ± 0.03	3.09 ± 0.22	1.80 ± 0.05
	Harmalol	0.26 ± 0.00	0.27 ± 0.02	0.25 ± 0.00
	Harmaline	0.19 ± 0.01	0.40 ± 0.00	0.26 ± 0.01
*P. viridis* + *P. harmala*	DMT	4.30 ± 0.08	3.94 ± 1.33	4.56 ± 0.15
	THH	ND	ND	ND
	Harmol	ND	ND	ND
	Harmine	1.64 ± 0.01	6.70 ± 0.12	3.05 ± 0.10
	Harmalol	0.37 ± 0.01	0.38 ± 0.01	0.34 ± 0.04
	Harmaline	4.62 ± 0.02	8.93 ± 0.04	4.05 ± 0.05
*M. hostilis* + *B. caapi*	DMT	9.07 ± 0.04	5.52 ± 0.09	7.36 ± 0.05
	THH	2.89 ± 0.21	2.37 ± 0.11	2.45 ± 0.04
	Harmol	0.28 ± 0.02	0.24 ± 0.01	0.22 ± 0.00
	Harmine	4.02 ± 0.04	10.34 ± 0.07	7.69 ± 0.27
	Harmalol	ND	ND	ND
	Harmaline	0.28 ± 0.01	0.42 ± 0.01	0.29 ± 0.01
*M. hostilis* + *P. harmala*	DMT	9.65 ± 0.12	4.76 ± 0.07	6.68 ± 0.16
	THH	0.89 ± 0.01	ND	ND
	Harmol	ND	ND	ND
	Harmine	4.41 ± 0.07	10.92 ± 0.23	9.38 ± 0.05
	Harmalol	0.60 ± 0.03	0.87 ± 0.01	0.63 ± 0.04
	Harmaline	11.45 ± 0.20	16.96 ± 0.11	12.08 ± 0.02

ND-not detected.

**Table 3:** Cell viability after exposure to extracts. The values are expressed as mean ± SD.

Samples	Cell Viability (%)
*P. viridis* Crude	156.01 ± 27.31
*P. viridis* Digested	128.85 ± 9.03
*B. caapi* Crude	95.92 ± 1.83
*B. caapi* Digested	113.66 ± 11.59
*P. harmala* Crude	171.46 ± 28.88
*P. harmala* Digested	117.62 ± 3.59
*M. hostilis* Crude	148.28 ± 14.18
*M. hostilis* Digested	96.04 ± 12.23
Commercial mixture Crude	101.50 ± 13.25
Commercial mixture Digested	79.52 ± 0.93
*P. viridis* + *B. caapi* Crude	148.07 ± 26.83
*P. viridis* + *B. caapi* Digested	103.74 ± 3.43
*P. viridis* + *P. harmala* Crude	162.55 ± 15.63
*P. viridis* + *P. harmala* Digested	127.75 ± 9.97
*M. hostilis* + *B. caapi* Crude	126.61 ± 16.39
*M. hostilis* + *B. caapi* Digested	118.50 ± 1.59
*M. hostilis* + *P. harmala* Crude	138.41 ± 17.63
*M. hostilis* + *P. harmala* Digested	120.70 ± 3.12

**Table 4:** TEER values obtained before and after incubation with the extracts. The values are expressed as mean ± SD. Statistically significant values were considered if *p* < 0.05 (*).

Samples	TEER (Ω cm^2^)		
	Before	After	*p*-Value
Control	990 ± 31.11	1034 ± 31.11	0.293
*P. viridis* Crude	1298 ± 155.56	1628 ± 207.94	0.239
*P. viridis* Digested	1518 ± 93.34	2046 ± 155.56	0.054
*B. caapi* Crude	1166 ± 155.56	1408 ± 110.73	0.146
*B. caapi* Digested	1232 ± 134.42	1276 ± 116.41	0.317
*P. harmala* Crude	1386 ± 217.79	1408 ± 124.45	0.913
*P. harmala* Digested	1188 ± 186.68	1496 ± 110.73	0.107
*M. hostilis* Crude	1254 ± 155.56	1298 ± 31.11	0.733
*M. hostilis* Digested	1584 ± 177.82	1496 ± 116.41	0.112
Commercial mixture Crude	1694 ± 155.56	1716 ± 232.83	0.754
Commercial mixture Digested	1232 ± 44.00	1232 ± 25.40	0.643
*P. viridis* + *B. caapi* Crude	1364 ± 186.68	1496 ± 0.00	0.423
*P. viridis* + *B. caapi* Digested	1166 ± 93.34	1386 ± 31.11	0.087
*P. viridis* + *P. harmala* Crude	1415 ± 93.34	1408 ± 91.59	0.936
*P. viridis* + *P. harmala* Digested	1100 ± 141.44	1144 ± 116.41	0.795
*M. hostilis* + *B. caapi* Crude	1232 ± 76.21	1276 ± 25.40	0.189
*M. hostilis* + *B. caapi* Digested	1188 ± 248.90	1408 ± 127.02	0.619
*M. hostilis* + *P. harmala* Crude	1254 ± 93.34	1430 ± 31.11	0.127
*M. hostilis* + *P. harmala* Digested	1232 ± 177.82	1452 ± 0.00	0.246

**Table 5:** Percentage of permeability of Caco-2 cells after incubation with the extracts. The values are expressed as mean ± SD. Statistically significant values were considered if *p* < 0.05 (*).

Samples	Permeability (%)	*p*-Value
Control	16.94 ± 2.35	-
*P. viridis* Crude	19.59 ± 3.00	0.281
*P. viridis* Digested	13.49 ± 1.03	0.165
*B. caapi* Crude	16.79 ± 0.14	0.879
*B. caapi* Digested	16.11 ± 0.49	0.823
*P. harmala* Crude	15.01 ± 0.46	0.462
*P. harmala* Digested	17.97 ± 1.37	0.523
*M. hostilis* Crude	14.38 ± 0.72	0.322
*M. hostilis* Digested	14.10 ± 0.41	0.267
Commercial mixture Crude	19.88 ± 2.84	0.383
Commercial mixture Digested	16.13 ± 1.83	0.865
*P. viridis* + *B. caapi* Crude	16.42 ± 0.40	0.959
*P. viridis* + *B. caapi* Digested	16.03 ± 1.50	0.463
*P. viridis* + *P. harmala* Crude	13.81 ± 0.49	0.225
*P. viridis* + *P. harmala* Digested	13.47 ± 1.85	0.283
*M. hostilis* + *B. caapi* Crude	13.07 ± 1.89	0.139
*M. hostilis* + *B. caapi* Digested	13.18 ± 0.16	0.074
*M. hostilis* + *P. harmala* Crude	12.40 ± 1.64	0.069
*M. hostilis* + *P. harmala* Digested	11.65 ± 1.79	0.058

**Table 6:** Concentration of the main compounds of Ayahuasca in the aliquots collected after the different incubation times with the crude extract (Mean μg/mL Extract) ± SD.

Samples	Compounds	Time		
		1 h	2 h	4 h
*P. viridis*	DMT	0.50 ± 0.01	0.89 ± 0.21	1.22 ± 0.05
*B. caapi*	THH	ND	0.66 ± 0.02	0.59 ± 0.06
	Harmol	ND	ND	ND
	Harmine	0.33 ± 0.03	1.00 ± 0.01	1.35 ± 0.03
	Harmalol	ND	ND	ND
	Harmaline	ND	ND	ND
*P. harmala*	THH	ND	ND	0.72 ± 0.12
	Harmol	ND	0.19 ± 0.02	ND
	Harmine	2.09 ± 0.02	4.41 ± 0.23	5.90 ± 0.04
	Harmalol	ND	ND	ND
	Harmaline	3.65 ± 0.06	5.71 ± 0.60	8.55 ± 0.17
*M. hostilis*	DMT	ND	1.18 ± 0.10	1.42 ± 0.06
Commercial mixture	DMT	ND	0.55 ± 0.04	0.73 ± 0.04
	THH	ND	ND	ND
	Harmol	ND	ND	ND
	Harmine	ND	ND	ND
	Harmalol	ND	ND	ND
	Harmaline	ND	ND	ND
*P. viridis* + *B. caapi*	DMT	0.16 ± 0.01	0.47 ± 0.06	0.63 ± 0.07
	THH	0.65 ± 0.02	0.78 ± 0.06	0.93 ± 0.04
	Harmol	ND	ND	ND
	Harmine	ND	0.42 ± 0.06	0.76 ± 0.04
	Harmalol	ND	ND	ND
	Harmaline	ND	ND	ND
*P. viridis* + *P. harmala*	DMT	0.33 ± 0.03	1.01 ± 0.16	1.10 ± 0.07
	THH	ND	ND	ND
	Harmol	ND	ND	ND
	Harmine	0.35 ± 0.02	0.39 ± 0.01	0.78 ± 0.04
	Harmalol	ND	ND	ND
	Harmaline	0.31 ± 0.00	0.67 ± 0.09	0.76 ± 0.03
*M. hostilis* + *B. caapi*	DMT	0.69 ± 0.07	1.67 ± 0.11	1.87 ± 0.04
	THH	0.74 ± 0.08	0.53 ± 0.03	0.65 ± 0.07
	Harmol	ND	ND	ND
	Harmine	0.98 ± 0.11	1.64 ± 0.11	2.42 ± 0.08
	Harmalol	ND	ND	ND
	Harmaline	ND	ND	ND
*M. hostilis* + *P. harmala*	DMT	0.29 ± 0.01	0.72 ± 0.04	1.24 ± 0.18
	THH	ND	ND	ND
	Harmol	ND	ND	ND
	Harmine	0.47 ± 0.02	1.07 ± 0.10	1.63 ± 0.27
	Harmalol	ND	ND	ND
	Harmaline	0.55 ± 0.08	1.07 ± 0.14	1.42 ± 0.22

ND-not detected.

**Table 7:** Concentration of the main compounds of Ayahuasca in the aliquots collected after the different incubation times with the digested extract (Mean μg/mL) ± SD.

Samples	Compounds	Time		
		1 h	2 h	4 h
*P. viridis*	DMT	0.73 ± 0.00	1.48 ± 0.02	1.99 ± 0.03
*B. caapi*	THH	ND	ND	ND
	Harmol	ND	ND	ND
	Harmine	ND	ND	1.14 ± 0.03
	Harmalol	ND	ND	ND
	Harmaline	ND	ND	ND
*P. harmala*	THH	ND	ND	ND
	Harmol	ND	ND	ND
	Harmine	1.83 ± 0.01	3.08 ± 0.09	4.19 ± 0.03
	Harmalol	ND	ND	ND
	Harmaline	3.81 ± 0.13	4.75 ± 0.12	5.63 ± 0.08
*M. hostilis*	DMT	ND	1.61 ± 0.07	1.90 ± 0.02
Commercial mixture	DMT	ND	0.61 ± 0.02	0.67 ± 0.02
	THH	ND	ND	ND
	Harmol	ND	ND	ND
	Harmine	ND	ND	ND
	Harmalol	ND	ND	ND
	Harmaline	ND	ND	ND
*P. viridis* + *B. caapi*	DMT	ND	0.50 ± 0.07	0.48 ± 0.01
	THH	ND	ND	ND
	Harmol	ND	ND	ND
	Harmine	ND	ND	0.70 ± 0.02
	Harmalol	ND	ND	ND
	Harmaline	ND	ND	ND
*P. viridis* + *P. harmala*	DMT	ND	0.65 ± 0.00	0.86 ± 0.03
	THH	ND	ND	ND
	Harmol	ND	ND	ND
	Harmine	ND	1.02 ± 0.01	1.26 ± 0.03
	Harmalol	ND	ND	ND
	Harmaline	0.74 ± 0.00	0.99 ± 0.01	1.26 ± 0.01
*M. hostilis* + *B. caapi*	DMT	0.78 ± 0.01	1.74 ± 0.01	2.11 ± 0.02
	THH	ND	ND	ND
	Harmol	ND	ND	ND
	Harmine	0.77 ± 0.00	1.96 ± 0.01	2.54 ± 0.64
	Harmalol	ND	ND	ND
	Harmaline	ND	ND	ND
*M. hostilis* + *P. harmala*	DMT	0.60 ± 0.03	1.57 ± 0.06	1.86 ± 0.02
	THH	ND	ND	ND
	Harmol	ND	ND	ND
	Harmine	0.85 ± 0.03	2.38 ± 0.09	3.34 ± 0.04
	Harmalol	ND	ND	ND
	Harmaline	1.10 ± 0.02	2.21 ± 0.01	3.02 ± 0.03

ND-not detected.

## Data Availability

The data presented in this study are available on request from the corresponding authors.

## Sample Availability

Not available.
